# Patterns of pre-treatment drug abuse, drug treatment history and characteristics of addicts in methadone maintenance treatment in Iran

**DOI:** 10.1186/1477-7517-9-18

**Published:** 2012-06-07

**Authors:** Hajar Shekarchizadeh, Hamed Ekhtiari, Mohammad R Khami, Jorma I Virtanen

**Affiliations:** 1 Community Oral Health Department, School of Dentistry, Tehran University of Medical Sciences, Tehran, Iran; 2 Department of Oral Public Health, University of Helsinki, Helsinki, Finland; 3 Translational Neuroscience Program, Institute for Cognitive Science Studies, Tehran, Iran; 4 Neurocognitive Laboratory, Iranian National Center for Addiction Studies, Tehran University of Medical Sciences, Tehran, Iran; 5 Department of Public Health, University of Helsinki, Helsinki, Finland; 6 Department of Community Dentistry, University of Oulu, P.O.Box 5281, FI-90014, Oulu, Finland

**Keywords:** Addiction, Opiates, Methadone Maintenance, Socioeconomic

## Abstract

**Background:**

Opiates are the main drugs of abuse, and Methadone Maintenance Treatment (MMT) is the most widely administered drug addiction treatment program in Iran. Our study aimed to investigate patterns of pre-treatment drug abuse, addiction treatment history and characteristics of patients in MMT in Tehran.

**Methods:**

We applied a stratified cluster random sampling technique and conducted a cross-sectional survey utilizing a standard patient characteristic and addiction history form with patients (n = 810) in MMT. The Chi-square test and *t*-test served for statistical analyses.

**Results:**

A clear majority of the participants were men (96%), more than 60% of whom were between 25 and 44 years of age, educated (89% had more than elementary education), and employed (>70%). The most commonly reported main drugs of abuse prior to MMT entry were opium (69%) and crystalline heroin (24%). The patients’ lifetime drug experience included opium (92%), crystalline heroin (28%), cannabis (16%), amphetamines (15%), and other drugs (33%). Crystalline heroin abusers were younger than opium users, had begun abusing drugs earlier, and reported a shorter history of opiate addiction.

**Conclusion:**

Opium and crystalline heroin were the main drugs of abuse. A high rate of addiction using more dangerous opiate drugs such as crystalline heroin calls for more preventive efforts, especially among young men.

## Introduction

Drug abuse is among the most challenging and costly health problems that lead to a broad range of physical, mental, and psychiatric consequences, and poses a huge public health problem worldwide [1]. Opioids are the third most widely used group of drugs after cannabis and amphetamine-type stimulants. Among opioids, opiates are the most problematic and harmful substances consumed by 12 to 21 million people worldwide. The highest consumption rate of opiates is in countries in Southwest Asia [2].

Iran, located along the opium trade route, has the highest rate of opiate drug use worldwide [3]. Based on the most recent national survey of drug abuse in Iran, the number of addicts in need of treatment services exceeds 1.2 million [4]. Opiates, mostly opium and crystalline heroin (heroin hydrochloride) [5], are the main drugs of abuse [6-8]. Recent years have seen a substantial shift from opium use towards using crystalline heroin: some new official estimates show that as many as 40% of opiate users consume opium, whereas the remaining users mostly consume different forms of heroin [2].

Generally, more than 80% of drug treatment admissions in Iran include opiate addicts seeking treatment [2]. Of various treatment methods, Methadone Maintenance Treatment (MMT) is considered the treatment of choice. According to Mokri and Schottenfeld [9], approximately 700 centers offered MMT to addicts in 2007. The treatment success among this group of addicts varies, but a multi-center study revealed a six-month retention rate of 23%, often requiring repeated treatment episodes [10].

Effective preventive care, treatment planning, and policy-making requires knowledge of characteristics and background factors regarding drug addicts. Limited published data are available on patients seeking treatment in addiction treatment centers in Iran. Thus, this study aimed to investigate patterns of pre-treatment drug abuse, addiction treatment history, and background characteristics of patients in MMT attending addiction treatment centers in Tehran.

## Materials and Methods

### Subjects

This study was a cross-sectional study of drug users attending methadone maintenance programs. The target population comprised all drug users attending methadone maintenance programs in addiction treatment centers in Tehran, Iran, in 2011. Based on the list of MMT clinics received from the Ministry of Health, more than 95% of the 160 addiction treatment centers in the city were private clinics. Thus, we focused on the clinics responsible for the major part of MMT and excluded the public centers from the study. We used a stratified cluster random sampling technique to cover the three main socioeconomic regions of the city: North, Center, and South.

Taking into account the proportion of clinics in each of the three strata, clinics were randomly selected as clusters in each study area. Based on the tariffs determined by the Ministry of Health in Iran, all private addiction treatment centers receive the same monthly fee from their patients. Thus, variation between the clinics in terms of the patient costs is minor. The region of the clinics makes use of the patients’ living area as an indicator of their socioeconomic status. After contacting the heads of the clinics and introducing the study, we inquired about their willingness to participate. Among the first randomly selected eight clinics two did not agree to participate, thus two substitutes were randomly selected. Totally, two clinics in the North, and three clinics in both the Center and South areas participated in the survey. At these eight centers, patients receiving MMT were asked to participate. All these patients met DSM-IV criteria for opioid dependence [11]. Altogether 810 patients participated in the study from January to May 2011 (response rate = 72%).

### Face-to-face interviews

Three trained interviewers conducted structured interviews with the participants. Prior to the study, the interviewers received special training and agreed on the content of the interview, the organization of the clinics, and how to communicate with the patients. In the clinics, the interviewers used a standard patient characteristic form commonly used as a framework in addiction studies in Iran to conduct structured ten-minute in-person interviews. The interviewers requested information on patients’ socioeconomic characteristics (age, gender, years of education, marital status, and job status), addiction history prior to treatment entry: all drugs used for at least one month in their lives, routes of abuse, the main drug of abuse (the most problematic drug, which was the main cause for treatment entry), age when the drug abuse began, the duration of addiction, and the total amount of money spent on drugs in the most recent month of abuse. In addition, the interviewers also inquired about the patients’ MMT history: their previous methadone treatments, the duration of current treatment, and the patients’ daily dose of received methadone.

The interviewers conducted the interviews in collaboration with the clinic nurse, who encouraged the patients to participate. Patients usually spent some time in the waiting room before receiving their medication or visiting their doctor, thus providing a good opportunity for the interview. The interviewer explained the study to the patients individually and inquired about their willingness to participate. After the interview, each patient received a toothbrush and toothpaste as a small gift. The data collection in each clinic continued to provide the monthly turnover of patients in the clinic based on the report provided by the clinic personnel.

### Ethical approval

The data collection was carried out using an anonymous patient characteristic form which aimed to provide as much confidentiality as possible. The study was voluntary, and all respondents provided their written informed consent. The Tehran University of Medical Sciences Ethics Committee approved the study.

### Statistical analysis

The data were transferred from the paper forms to a digital format. We performed statistical data analysis using the Statistical Package for the Social Sciences (SPSS for Windows, version 18), and analyzed any differences in frequencies using the Chi-square test; the *t*-test served to compare the means (level of significance < 0.05).

## Results

More than 95% of the participants receiving MMT were men, whereas women comprised 4% of the patients. The socioeconomic background of the participants appears in Table 1. The mean age of the patients was 40.5 years (SD 11.5; range 20–86), a majority (> 60%) of whom were between 25 and 44 years of age, married (70%), and employed (> 70%). Over 60% of the female participants were homemakers. As many as 89% of the patients had more than elementary school education (65% high school and 24% university education), whereas the others had an elementary school background (9%) or were illiterate (2%).

**Table 1:** Characteristics and main drug of abuse among patients receiving MMT (n = 810) in Tehran

**Variable**	**Male**		**Female**		**Total**	
	**N**	**%**	**N**	**%**	**N**	**%**
**Age**						
18–24	26	3	4	12	30	4
25–34	263	34	11	33	274	34
35–44	234	30	4	12	238	29
45–54	151	19	10	30	161	20
55–64	81	10	4	12	85	11
65≤	22	3	−	−	22	3
Total	777		33		810	
**Marital status**						
Married	541	70	23	70	564	70
Single	164	21	4	12	168	21
Widow	7	1	2	6	9	1
Divorced	64	8	4	12	68	8
Total	776		33		809	
**Education status**						
Illiterate	12	2	5	16	17	2
Elementary school	68	9	2	6	70	9
High school	500	66	19	59	519	65
University	183	24	6	19	189	24
Total	763		32		795	
**Job status (in the last 3 months)**						
Full time job	424	55	4	13	428	53
Unemployed	115	15	4	13	119	15
Student	10	1	−	−	10	1
Retired	74	10	1	3	75	9
Part time job	153	20	3	9	156	19
Homemaker	−	−	20	63	20	3
Total	776		32		808	
**Main drug of abuse**						
Opium	534	69	19	59	553	69
Crystalline Heroin	180	23	11	34	191	24
Other drugs	60	8	2	6	62	8
Total	774		32		806	

The most commonly reported main drug of abuse prior to treatment was opium (69%), followed by crystalline heroin (24%). Other drugs served as the main drug of abuse for about 8% of the participants: brown heroin base (3%), Norjizak (vial heroin) (0.5%) [12], amphetamines (1.2%), methadone (0.6%), cocaine (0.1%), tranquilizers (0.6%), and alcohol (0.1%) among others. In terms of their lifetime history of drug abuse, more than 90% of the participants reported using opium, 28% crystalline heroin, 16% cannabis, 15% amphetamines, and 33% other drugs for at least one month during their addiction period. Smoking and oral routes were the most prevalent routes of abuse reported by 79% and 48% of the participants, respectively. Snorting and injection were reported as routes of abuse by 5% of the participants. Table 2 presents the demographic characteristics of the participants according to the three major drug abuse groups. Crystalline heroin abuse was more prevalent in patients under 35 than was opium abuse (*p* < 0.001; df = 1), and crystalline heroin users were more educated than were opium users (mean 11.6 years vs. 10.6 years; *p* < 0.05).

**Table 2:** Characteristics of patients receiving MMT (n = 810) in Tehran according to their main drug of abuse

**Variable**	**Opium**	**Crystalline Heroin**	**Other Drugs**	**Total**
	**(N %)**	**(N %)**	**(N %)**	**(N %)**
**Gender**				
Male	534 (97)	180 (94)	60 (97)	774 (96)
Female	19 (3)	11 (6)	2 (3)	32 (4)
Total	553	191	62	806
Age*				
18–24	9 (2)	20 (11)	1 (2)	30 (4)
25–34	140 (25)	100 (52)	32 (52)	272 (34)
35–44	161 (29)	57 (30)	20 (32)	238 (30)
45–54	140 (25)	14 (7)	5 (8)	159 (20)
55–64	81 (15)	−	4 (7)	85 (11)
65≤	22 (4)	−	−	22 (3)
Total	553	191	62	806
Education status*				
Illiterate	14 (3)	3 (2)	−	17 (2)
Elementary school	60 (11)	6 (3)	4 (7)	70 (9)
High school	347 (64)	127 (68)	42 (69)	516 (65)
University	121 (22)	52 (28)	15 (25)	188 (24)
Total	542	188	61	791
Job status (in the last 3 months)*				
Full time job	305 (55)	93 (49)	28 (45)	426 (53)
Unemployed	64 (12)	36 (19)	19 (31)	119 (15)
Student	4 (1)	6 (3)	−	10 (1)
Retired	70 (13)	2 (1)	3 (5)	75(9)
Part time job	97 (18)	47 (25)	11 (18)	155 (19)
Homemaker	12 (2)	6 (3)	1 (2)	19 (2)
Total	552	190	62	804

*Variables with significant differences, *p* < 0.05.

A summary of the addiction history of the patients in the three main drug abuse groups appears in Table 3. An overwhelming majority of addicts seeking treatment had used drugs for at least one year, whereas few (3%) reported a history of addiction to drugs of less than one year. The mean duration of addiction among the patients was 10.8 years (SD 7.9). Generally, crystalline heroin abusers began abusing drugs earlier than did opium users (mean 21.2 years vs. 26.2 years; *p* < 0.001); the starting age for 76% of crystalline heroin abusers was under 25 years. The duration of addiction among crystalline heroin users was shorter than among opium users (mean 8.7 years vs. 11.6 years; *p* < 0.001). Opium users reported smoking (77%) and oral route (58%) as the most common routes of abuse prior treatment. Among crystalline heroin abusers smoking (94%) was the most prevalent route of use (Table 4).

**Table 3:** Addiction history of patients receiving MMT (n = 810) in Tehran according to their main drug of abuse

**Variable**	**Opium (N %)**	**Crystalline heroin (N %)**	**Other drugs (N %)**	**Total (N %)**
Age of starting drug abuse (years)*				
≤ 17	55 (10)	43 (23)	11 (18)	109 (14)
18–24	210 (38)	102 (53)	31 (51)	343 (43)
25–34	187 (34)	43 (23)	14 (23)	244 (31)
35 ≤	97 (18)	3 (2)	5 (8)	105 (13)
Total (N)	549	191	61	801
**Mean (SD)**	26.2 (8.6)	21.2 (5.3)	23 (7.8)	24.8 (8.2)
Duration of addiction (years)*				
< 1	13 (2)	6 (3)	4 (7)	23 (3)
1–5	144 (26)	61 (33)	18 (30)	223 (28)
6–10	156 (29)	64 (34)	13 (21)	233 (29)
11 ≤	233 (43)	56 (30)	26 (43)	315 (40)
Total (N)	546	187	61	794
**Mean (SD)**	11.6 (8.4)	8.7 (6.2)	9.9 (7.8)	10.8 (7.9)
Duration of current treatment (months)*				
< 1	67 (12)	21 (11)	5 (8)	93 (12)
1–5.9	144 (26)	61 (32)	12 (20)	217 (27)
6–11.9	126 (23)	47 (25)	4 (7)	177 (22)
12–23.9	112 (20)	32 (17)	10 (16)	154 (19)
24 ≤	102 (19)	29 (15)	30 (49)	161 (20)
Total (N)	551	190	61	802
**Mean (SD)**	12.8 (15.6)	10.3 (10.7)	25.2 (25.5)	13.1 (16)
Number of previous MMT admissions*				
0	454 (82)	139 (73)	48 (77)	641 (80)
1 ≤	97 (18)	52 (27)	14 (23)	163 (20)
Total (N)	551	191	62	804
Dosage of methadone (mg)*				
≤ 30 mg	193 (36)	41 (22)	16 (26)	250 (32)
35–65 mg	189 (35)	58 (31)	18 (30)	265 (34)
70 mg ≤	158 (29)	90 (48)	27 (44)	275 (35)
Total (N)	540	189	61	790
**Mean (SD)**	57.9 (51.6)	70.5 (46)	80.7 (75)	62.6 (52.9)
Drug cost in the last month before treatment entry ($)*				
< 50	93 (18)	7 (4)	12 (20)	112 (15)
50–99	133 (25)	6 (3)	7 (12)	146 (19)
100–199	198 (38)	34 (18)	10 (16)	242 (31)
200–499	85 (16)	97 (52)	15 (25)	197 (26)
500 ≤	17 (3)	43 (23)	17 (28)	77 (10)
Total (N)	526	187	61	774
**Mean (SD)**	133 (155)	381 (372)	303 (278)	206 (259)

*Variables with significant differences, *p* < 0.05.

**Table 4:** Routes of using drugs prior to treatment among MMT patients (n = 810) in Tehran according to their main drug of abuse

**Variable**	**Opium (N %)**	**Crystalline heroin (N %)**	**Other drugs (N %)**	**Total (N %)**
**Smoking route**				
Yes	425 (77)	180 (94)	29 (48)	634 (79)
No	126 (23)	11 (6)	32 (52)	169 (21)
Total (N)	551	191	61	803
**Snorting (Sniffing) route**				
Yes	4 (1)	14 (7)	10 (16)	28 (4)
No	547 (99)	177 (93)	51 (84)	775 (96)
Total (N)	551	191	61	803
**Oral route**				
Yes	322 (58)	33 (17)	32 (53)	387 (48)
No	229 (42)	158 (83)	29 (47)	416 (52)
Total (N)	551	191	61	803
**Injection route**				
Yes	3(1)	19 (10)	16 (26)	38 (5)
No	548 (99)	172 (90)	45 (74)	765 (95)
Total	551	191	61	803

For the vast majority (80%) of patients, the treatment program they were already in was their first MMT episode (Table 3). The mean duration of current treatment was 13.1 months (SD 16) and it had lasted at least six months for most of them (61%). The dose of methadone participants received ranged from 1.20 mg to 500 mg (mean 62.6; SD 52.9; median 55.0). The duration of current treatment was shorter (mean 10.3 months vs. 12.8 months; *p* < 0.05) and the dosage of methadone was higher among crystalline heroin users than among opium users (mean 70.5 mg vs. 57.9 mg; *p* < 0.05).

About two-thirds of the patients (67%) reported spending at least USD 100 per month (mean USD 200) on their drugs during the last month prior to treatment (Table 3). Crystalline heroin users reported the highest monthly drug expenses: 75% of them spent at least USD 200 per month on their drugs whereas fewer than 20% of opium users spent an equivalent amount on theirs.

## Discussion

Our study shows that in Tehran, patients receiving MMT predominantly used opium and crystalline heroin, whereas other drugs were the main drugs for only a minority of MMT patients. Generally, the clear majority of participants receiving MMT were young, educated, married, and employed men.

According to the world drug report, in 2009, between 149 and 272 million people between the ages of 15 and 64 consumed illegal drugs, of whom about 10% to 20% were considered dependent and regular drug users [2]. Patterns of drug dependency vary across countries: Iran, with more than one million regular drug users, has the world’s highest rate of dependence on opiate drugs [3,4].

Tehran, as the capital of Iran and with a population exceeding 12 million, comprises a considerable section of the population. This study was conducted on MMT patients of private outpatient treatment centers in Tehran. Because public centers are so few, our sample provides a good overview of patients participating in MMT programs. MMT is the main treatment for opiate users available at official outpatient medical addiction treatment centers; consequently, this sample sheds light on the majority of opiate addiction treatment seekers who were referred to this official network.

Opium seems still to be the drug of choice among Iranian addicts given that most studies in Iran acknowledged the higher frequency of addiction to opium than to other drugs [6,8]. Our results are similar to the findings of a previous study of a group of drug addicts seeking treatment in a general practice in Shiraz, which reported opium and heroin as the drugs most commonly abused [7]. The most recent Iranian rapid situation assessment of drug abuse in 2007 reported opium, crystalline heroin, and heroin to be the three most prevalent drugs previously abused by those attending treatment centers and in the general population [4]. Similar findings have also been reported in a multi-center study of seven outpatient centers in four Iranian cities [10]. The high prevalence of the history of opium use in our study compared to the estimates in the World Drug Report 2011 might be due to seeking treatment or less stigmatized self-report of using opium instead of heroin [2]. Only 1% of the patients reported amphetamines as their main drug of abuse, but as many as 15% reported using this drug for at least one month in their lifetime prior to treatment. Since amphetamines are easily available in the Iranian drug market in comparison with other stimulants like cocaine [13], and that they may alleviate some of methadone side effects, they can cause a serious threat for the treatment process in the MMT clinics in Iran.

Crystalline heroin users were younger than opium users, and most of them had begun using drugs at younger ages. Moreover, the duration of addiction among crystalline heroin users tended to be shorter than among opium users. The amount of money most of the participants had spent on drugs was higher than the monthly MMT fee in these clinics (i.e. USD 80–90). This monthly treatment fee consists of a small proportion of the average monthly income of an urban family in Tehran http://www.cbi.ir. Crystalline heroin users had spent more money on their drugs during their last month of addiction than had opium addicts. Although a majority of the participants reported being under some kind of treatments earlier, most of them were receiving MMT for the first time. Overall, about 60% of the patients had been in MMT for more than six months. The mean daily dose of methadone among crystalline heroin users was higher than among opium users, but, it was in the recommended range of 60 to 120 mg [14-16]. Although the mean daily dose of methadone was in the recommended range, as much as 30% of the patients received up to 30 mg methadone. In Iran, methadone is usually prescribed in syrup or tablet forms. In our study the proportion of methadone in syrup and tablet forms among the MMT patients was about the same.

In line with previous findings, drug abuse is strongly related to male gender, particularly in the Eastern Mediterranean region [17-21]. Little is known about female addicts in the Middle East, and no comprehensive study on the prevalence of drug abuse among Iranian women exists. Consequently, it is unclear whether the low proportion of female participants in our study was due to a limited number of female addicts in general or their reluctance to seek professional help in treatment centers. Possible reluctance of women to give their consent to participate in the study was unlikely since the response rate among female participants (74%) was close to the overall response rate (72%).

In our study, most of the patients receiving MMT were fairly young. This is in line with a study by Day et al. [7] of drug abusers in a general practice in Shiraz, Iran. Another report of drug abuse among the general population in Kerman, however, found no significant association between drug abuse and age group [8]. Studies in other countries also show a higher prevalence of addiction among young people [17,18,21,22].

In contrast with other studies of general populations that reveal lower rates of addiction among more educated people [6,23], most of the MMT participants in our study had more than elementary school education. A study of drug abuse in Thailand reported somewhat similar findings among addicts in rural areas [21]. As a review article by Mokri [24] emphasized, most of the participants attending treatment centers in our study were also employed. Similar results emerged in another study of opioid-dependent patients seeking treatment in a major center in Shiraz [25]. In contrast, another study of opium use in a rural area of Iran found a significant association between opium use and unemployment [6]. On the other hand, in a population-based study of opium use in Fars province, opium use cut across all employment status groups [23]. The differences between the profile of MMT patients in our study and other studies in similar settings with general population of addicts reflect the diversity of the patients in terms of their socioeconomic status including education and employment. This indicates that in-treatment patients may be of higher socioeconomic level and may have more resources than the other addicts.

Bearing in mind that this study was a cross-sectional survey of patients attending outpatient addiction treatment centers, no causal inferences of underlying factors can be outlined. In addition, the findings from patients in MMT cannot be generalized to the whole population of drug users. One limitation of the study is that because of practical reasons only eight clinics were included in the study. Although the data are based on patients’ self-reported addiction history, we tried to decrease the likelihood of underreporting via good collaboration with the clinic staff. However, it has to be kept in mind that tendency towards “social desirability” among clients in a good therapeutic alliance to caregivers can be a source of under-reporting. This limitation is true for all studies regarding drug abuse history.

Despite these limitations, our study has several strengths. The stratified random sampling method outlined well patients seeking treatment in addiction treatment centers from all three main socioeconomic areas of the city. In addition to the broad coverage, the large sample size along with the high response rate in this marginalized target group bolsters the reliability of the results in this capital city of more than 12 million. It is worth mentioning that our response rate of 72% may be somewhat low, as it is based on the number of study participants among the total monthly turnover in the clinics, rather than on the number of patients asked to participate. Furthermore, by utilizing a structured interview instead of a self-administered questionnaire, we reduced the possibility of misunderstandings in this compromised group.

Our study provides valuable information about the characteristics of patients attending addiction treatment centers in Iran. Although Tehran represents a considerable proportion of the Iranian population, a national survey of all drug abusers would be necessary to obtain a more thorough understanding of the predisposing factors associated with illegal drug abuse.

## Conclusion

The present study has demonstrated that among Iranian former drug addicts attending addiction treatment centers, opium and crystalline heroin are the drugs of choice. The growing number of addicts utilizing more dangerous drugs such as crystalline heroin calls for more educational and preventive strategies targeting younger age groups in particular.

## Abbreviations

MMT, Methadone Maintenance Treatment.

## Competing interests

The authors declare that they have no competing interests.

## Authors’ contribution

All authors were involved in the study concept and design. HS carried out data collection. HS and JV performed statistical analyses and interpretation of the data. All authors participated in writing of manuscript. All authors read and approved the final manuscript.
